# A Cost-Effectiveness Analysis of a Program to Control Rheumatic Fever and Rheumatic Heart Disease in Pinar del Rio, Cuba

**DOI:** 10.1371/journal.pone.0121363

**Published:** 2015-03-13

**Authors:** David A. Watkins, Mercy Mvundura, Porfirio Nordet, Bongani M. Mayosi

**Affiliations:** 1 Division of General Internal Medicine, Department of Medicine, University of Washington, Seattle, Washington, United States of America; 2 Department of Medicine, Groote Schuur Hospital and the University of Cape Town, Cape Town, South Africa; 3 PATH, Seattle, Washington, United States of America; 4 Retired from the cardiovascular disease programme, World Health Organization, Geneva, Switzerland; Leibniz Institute for Prevention Research and Epidemiology (BIPS), GERMANY

## Abstract

**Background:**

Acute rheumatic fever (ARF) and rheumatic heart disease (RHD) persist in many low- and middle-income countries. To date, the cost-effectiveness of population-based, combined primary and secondary prevention strategies has not been assessed. In the Pinar del Rio province of Cuba, a comprehensive ARF/RHD control program was undertaken over 1986 – 1996. The present study analyzes the cost-effectiveness of this Cuban program.

**Methods and Findings:**

We developed a decision tree model based on the natural history of ARF/RHD, comparing the costs and effectiveness of the 10-year Cuban program to a “do nothing” approach. Our population of interest was the cohort of children aged 5 – 24 years resident in Pinar del Rio in 1986. We assessed costs and health outcomes over a lifetime horizon, and we took the healthcare system perspective on costs but did not apply a discount rate. We used epidemiologic, clinical, and direct medical cost inputs that were previously collected for publications on the Cuban program. We estimated health gains as disability-adjusted life years (DALYs) averted using standard approaches developed for the Global Burden of Disease studies. Cost-effectiveness acceptability thresholds were defined by one and three times per capita gross domestic product per DALY averted. We also conducted an uncertainty analysis using Monte Carlo simulations and several scenario analyses exploring the impact of alternative assumptions about the program’s effects and costs. We found that, compared to doing nothing, the Cuban program averted 5051 DALYs (1844 per 100,000 school-aged children) and saved $7,848,590 (2010 USD) despite a total program cost of $202,890 over 10 years. In the scenario analyses, the program remained cost saving when a lower level of effectiveness and a reduction in averted years of life lost were assumed. In a worst-case scenario including 20-fold higher costs, the program still had a 100% of being cost-effective and an 85% chance of being cost saving.

**Conclusions:**

A 10-year program to control ARF/RHD in Pinar del Rio, Cuba dramatically reduced morbidity and premature mortality in children and young adults and was cost saving. The results of our analysis were robust to higher program costs and more conservative assumptions about the program’s effectiveness. It is possible that the program’s effectiveness resulted from synergies between primary and secondary prevention strategies. The findings of this study have implications for non-communicable disease policymaking in other resource-limited settings.

## Introduction

Acute rheumatic fever (ARF) and rheumatic heart disease (RHD) remain the most common cardiovascular condition in children and young adults in low- and middle-income countries [1], with high morbidity and mortality in endemic regions such as sub-Saharan Africa [2]. Disparities in RHD between developing countries are due in part to gaps in the implementation of ARF/RHD control programs [3]. Control strategies for RHD generally focus on one or more of three approaches: 1) primary prevention, i.e., appropriately managing streptococcal pharyngitis; 2) secondary prevention, i.e., finding cases of ARF/RHD and increasing rates of antibiotic prophylaxis; or 3) medical and surgical treatment of existing cases of RHD to prevent premature death [4].

Cost-effectiveness analysis is an important tool for decision makers seeking to implement ARF/RHD control measures, especially in settings where the disease burden is high but intervention coverage and public sector budgets are low [5]. Previously published economic evaluations on ARF/RHD have focused on one of two questions. First, several studies have compared clinical algorithms for managing pharyngitis (primary prevention) in, e.g., the United States [6, 7], and South Africa [8]. Secondly, other studies have sought to assess tradeoffs between alternative strategies for RHD prevention [5, 9, 10]. These studies generally place primary and secondary prevention strategies at odds with each other and often find secondary prevention (in isolation) to be more cost-effective [5, 10].

By contrast, the World Health Organization has recommended that ARF/RHD programs be comprehensive in scope and include scale up of primary and secondary prevention, community education, and surveillance activities [11]. Experience in the United States during the mid-20^th^ century [12] and more recently, in the Latin America and Caribbean region [13, 14] suggests that dramatic reductions in the incidence of ARF—and thus the burden of RHD—are feasible with concerted public health efforts. In particular, a 10-year program in the Pinar del Rio province of Cuba observed large reductions in the burden of ARF and RHD and medical expenditures on these conditions [14].

Despite these observations, the cost-effectiveness of comprehensive ARF/RHD control efforts has not been determined. At present, the controversies in the literature, e.g., whether primary prevention should be emphasized over secondary prevention [2, 5] and the role of echocardiography-based screening [10], have not considered the empirical data from countries that have successfully eliminated ARF and RHD through comprehensive control programs. The aforementioned experiences in the United States, Cuba, and Costa Rica suggest that a comprehensive program may be highly cost-effective or even cost saving, in contrast to program focusing only on secondary prevention. Hence a formal cost-effectiveness analysis of this type of program could have immediate applicability to policymakers in endemic regions. In the present study, we use previously published data from the Cuban program in Pinar del Rio province [14] to assess the cost-effectiveness of a comprehensive effort to control ARF/RHD.

## Materials and Methods

### Background and Setting

Pinar del Rio is a mostly urban province of 10,931 km^2^ in the western part of Cuba, with 721,800 population in 1996. Nordet et al. described in detail the design and implementation of a primary healthcare-oriented program to reduce the burden of ARF/RHD in the province [14]. The program included community and healthcare provider education and training around treatment of sore throat and prevention of ARF and RHD (primary prevention) as well as development of an ARF/RHD register and improvement of patient adherence to penicillin prophylaxis (secondary prevention). Prevalence of RHD in school-aged children was measured before and after the program, and they also recorded the incidence of new-onset and recurrent ARF in their province. The authors reported a progressive decline in the incidence of ARF and severity of RHD in the province over the study period, and the effects were sustained at follow-up six years after the program was completed [14].

### Modeling Approach

**Fig 1:**
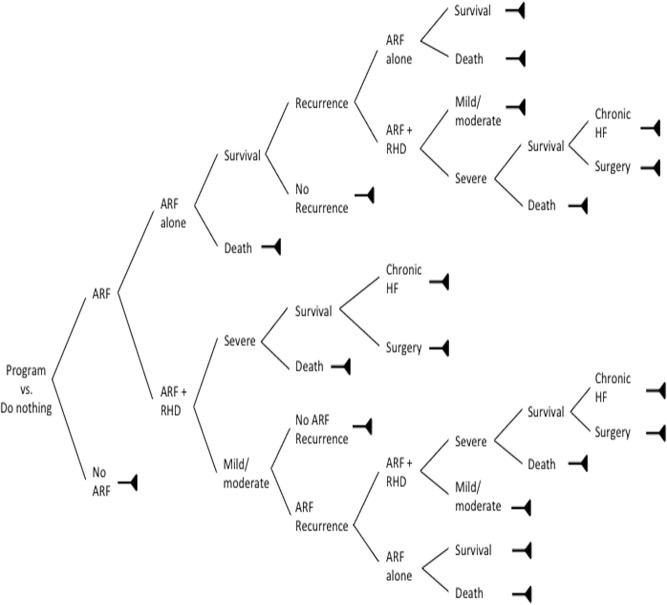
Decision analysis model used in the study.

We conducted a cost-utility analysis using disability-adjusted life years (DALYs) as the measure of the health burden of ARF and RHD. We compared the Pinar del Rio program with a “do nothing” approach, because at a national level, there was no change in mortality from ARF and RHD over the same time period [15], and similar RHD control efforts had not yet been scaled up in the rest of Cuba. Based on our clinical experience, we developed a decision tree to represent the various disease states associated with ARF and RHD (Fig. 1). We used this decision tree to estimate—for both the intervention and “do nothing” approaches—the total number of cases of ARF and RHD in each of these states.

For our model cohort, we used the population of children and young adults aged 5–24 years that were residing in Pinar del Rio in 1986, the program’s index year (n = 273,933). This allowed us to replicate the epidemiological data and program costs reported by Nordet et al. We chose the healthcare system (government) perspective on costs, reflecting the fact that Cuba’s economy is centrally planned and because no household costs were available. Our analytic horizon was the lifetime of the cohort, but as the major effects of the program occurred in the past, we chose not to discount costs and health outcomes.

### Epidemiologic and Clinical Inputs

Our decision tree incorporated the changes in ARF incidence that were measured by Nordet et al. [14] and Lopez [16] during the program. We also used age-standardized ARF and RHD mortality rates over 1986–1996 published by the Cuban Ministry of Health [15] to calculate case-fatality rates. For the do-nothing approach, we assumed constant incidence rates of ARF and RHD, because as mentioned previously, this was observed throughout the rest of Cuba during the study period. The key epidemiologic parameters are listed in Table 1. We estimated the rest of the transition probabilities in our decision tree using aggregated data on ARF and RHD morbidity reported by Nordet et al. and Lopez. The Supporting Information describes these calculations in more detail (S1 File) and provides a full list of transition probabilities (S1 Table).

**Table 1:** Main epidemiologic inputs to the model.

	Control	Intervention	Source
**Incidence of ARF**			
1986	12.2 / 100,000	12.2 / 100,000	Nordet et al.[14]
1987	12.2 / 100,000	7.4/ 100,000	Nordet et al.[14]
1988	12.2 / 100,000	2.6 / 100,000	Nordet et al.[14]
1989	12.2 / 100,000	2.7 / 100,000	Nordet et al.[14]
1990	12.2 / 100,000	2.8 / 100,000	Nordet et al.[14]
1991	12.2 / 100,000	3.0 / 100,000	Nordet et al.[14]
1992	12.2 / 100,000	3.2 / 100,000	Nordet et al.[14]
1993	12.2 / 100,000	4.1 / 100,000	Nordet et al.[14]
1994	12.2 / 100,000	1.9 / 100,000	Nordet et al.[14]
1995	12.2 / 100,000	2.0 / 100,000	Nordet et al.[14]
**ARF case-fatality rate** *	0.014	0.014	MINSAP[15]
**RHD case-fatality rate**			
With first ARF ** episode	0.357	0.000	Lopez[16]
With recurrent ARF **	0.444	0.000	Lopez[16]
With progression of RHD	0.385	0.000	Lopez[16]
**Probability of ARF recurrence**			
With history of ARF	0.567	0.176	Lopez[16]
With history of RHD	0.778	0.250	Lopez[16]
**Probability of requiring surgery**	0.071–0.154	0.044–0.060	
First presentation with RHD	0.071	0.000	Lopez[16]
Progression of RHD	0.154	0.000	Lopez[16]

Null case-fatality rates were varied in the uncertainty analysis (S1 Table).

* Estimated from published RHD mortality rates and calculated incidence rates.

** “RHD mortality” in this case reflects presence of severe carditis with ARF.

### Estimation of Program Costs

The RHD control program in Pinar del Rio involved several major components: administration, education, surveillance, and external evaluation. To estimate the time cost of the program, we used standard Cuban wage rates, converted and inflated to 2010 US dollars, for all staff and providers involved in the program (Ministry of Health—personal communication). We also included the cost of the technologies used in the prevalence studies and the media costs associated with the health education campaigns [16]. Finally, we calculated the cost of an external program evaluation that was conducted by the World Health Organization. The Supporting Information (S1 File, S2 Table) provides the details of the costing process.

### Estimation of Direct Medical Costs

Clinical care was and is still provided free of charge to Cuban citizens, so estimates of the direct costs of medical care are not readily available. In order to estimate the healthcare system cost of providing medical care, we used the prices of medical services that were charged to visitors of Cuba during the study period [16]. We defined major cost categories as those associated with hospitalization for 1) ARF, 2) mild-moderate RHD, 3) severe RHD leading to heart failure, and 4) severe RHD requiring surgical valve replacement. We also calculated lifetime medical costs including follow-up diagnostics, medications, and provider fees. We separately included the cost of secondary prophylaxis at 1986 adherence levels (control scenario, 68.3%) and 1996 adherence levels (intervention scenario, 96.9%). The final cost estimates are listed in Table 2; individual components are detailed in the Supporting Information (S3 Table).

**Table 2:** Costs associated with ARF and RHD in Pinar del Rio, Cuba.

**Direct medical costs**	
Cost of hospitalization per episode	
ARF alone	$1490
Mild/moderate RHD	$2139
Severe RHD	$6343
Lifetime cost of secondary prevention *	
ARF alone	$3205 – $6222
ARF with RHD	$7262 – $11,161
**Total lifetime costs** *	
ARF	$15,515 – $18,699
Mild-moderate RHD	$21,359 – $25,259
Severe RHD	$40,714 – $49,298
Surgery for RHD	$56,285 – $62,532
**Program costs (over a 10-year period)**	
Administrative costs	$43,372
Educational costs—health workers	$151,206
Educational costs—community	$1360
Survey cost—before intervention	$1796
Survey cost—after intervention	$4021
External evaluation costs	$1134
Total program costs	$202,890

All costs in 2010 US dollars. See main text for costing methods and sources.

* Value within range depends on age at initial diagnosis.

### Estimation of Disability-Adjusted Life Years

In order to account for differential health gains by age, as well as the differential improvement in utility experienced by patients with RHD of varying severity, we calculated all health outcomes in terms of DALYs. Age-specific years of life lost (YLL) were calculated using formulas developed for the Global Burden of Disease (GBD) studies [17, 18]. Years lived with disability (YLD) were calculated using GBD 2010 disability weights [19]. Consistent with prior GBD studies, we assumed the duration of chronic RHD was five years, followed by death.[17] Further details on the DALY calculations are described in the Supporting Information (S1 File).

### Uncertainty and Scenario Analyses

Although the original studies by Nordet et al. [14] and Lopez [16] reported point estimates of changes in disease severity and medical expenditures, we attempted to account for uncertainty associated with the clinical and cost data we used as inputs for our model. We thus calculated minimum and maximum values for all transition probabilities using ranges published in the literature or, more frequently, using standard error formulas for binomial variables. We varied the direct medical costs and program costs from 50% to 200% of the baseline estimate. To model the joint uncertainty in all of our parameters, we calculated the DALYs and costs in 1000 Monte Carlo simulations. For each simulation, we drew transition probabilities from beta distributions parameterized by their upper and lower bounds, and we drew costs from a random uniform distribution.

We identified three critical assumptions inherent in our model and thus tested their impact on our estimates in four scenario analyses. First, secular trends in RHD globally between 1990 and 2010 suggest that the yearly decline in the burden of RHD was approximately 2.5% [20], so we incorporated the same linear decline in ARF incidence in our “do nothing” approach. Second, there were no local data to support the five year average duration of RHD used in the GBD studies to calculate YLL [17], so we increased the duration to 20 years to understand the impact this would have on total YLL averted. Third because wages in Cuba during the study period were quite low (owing to the country’s unique economy) and the true economic cost of the program in a similar developing country setting in the present day might actually be much higher, we increased the program costs 20-fold. Fourth, we created a “worst case” scenario that simultaneously incorporated all three prior scenario analyses. Similar to the base case, we performed 1000 simulations to quantify uncertainty associated with each scenario analysis.

### Other Methods

As mentioned above, in the base case we used an exchange rate of 1 Cuban Pesos to 1 US dollar, which was the formal government exchange rate during the 1980s. We inflated all costs to 2010 US dollars using consumer price index information from the World Bank [21]. Because the cost analysis was conducted on a program in the past, we did not discount the cost estimates or DALYs. Based on WHO-CHOICE criteria, we considered the program very cost effective if the cost per DALY averted was less than the gross domestic product (GDP) per capita and cost effective if the cost per DALY averted was between one and three times the GDP per capita [22]. We also followed the WHO “best buy” framework and used a threshold cost of less than US$ 100 per DALY averted to represent a “cheap” intervention in the local context [23]. We used World Bank estimates of gross domestic product and Cuba’s 2010 population size to arrive at the following cost-effectiveness thresholds: less than US$ 5702 per DALY averted was “very cost effective,” and between US$ 5702 and US$ 17,106 per DALY averted was “cost-effective” [24]. Costs were estimated in Microsoft Excel 2011, and the decision tree as well as the uncertainty and scenario analyses were developed and run in R [25]. Code for the R simulations is available from the authors upon request.

### Ethical considerations

Because this analysis used only publicly available or previously published data, it was deemed “not human subjects research” according to the criteria of the University of Washington’s institutional review board. All authors had full access to the study data, reviewed the manuscript, and provided consent for publication. The authors declare no competing interests.

## Results

### Base case

In a cohort of 273,933 at-risk Cuban children, implementation of the ARF/RHD program averted 231 cases of ARF, 117 cases of RHD, and US$ 7,848,590 in total direct medical costs, the latter of which vastly outweighed the program investments of US$ 202,890. These health gains translated into 5051 DALYs averted, and because the program was cost saving, it dominated the do-nothing approach in all 1000 Monte Carlo simulations (Table 3).

### Scenario analyses

**Fig 2:**
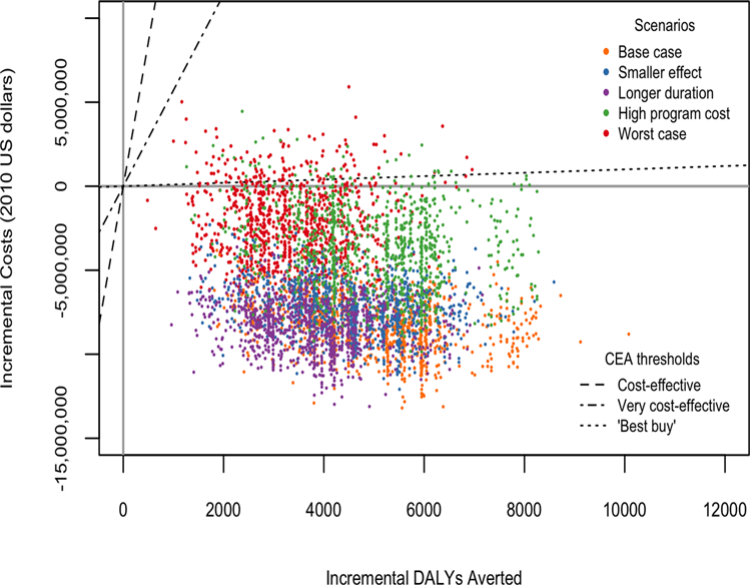
Uncertainty and scenario analyses: variation in incremental cost-effectiveness ratio of the ARF/RHD program compared to a “do-nothing” strategy. All costs are in 2010 US dollars.

We found that modeling the global trend as the counterfactual (do-nothing) trend and increasing the average duration of RHD did not substantially alter the results. When the program costs were increased 20-fold and when the worst case scenario was modeled, the program was not cost saving in all simulations, yet it remained “very cost effective” compared with the do-nothing approach in all simulations. Table 3 and Fig. 2 present these results as a range of cost-effectiveness ratios (ICERs) to aid in the interpretation of scenarios where the program was not cost saving.

**Table 3:** Program cost-effectiveness, including results of uncertainty analysis in five scenarios.

Scenario	ICER (95% interval)	Probability cost saving	Probability “best buy”	Probability very cost-effective	Probability cost-effective
**Base case**	-$1728	100%	100%	100%	100%
	(-$3154;-$942)				
**Lower program effectiveness**	-$1615	100%	100%	100%	100%
	(-$3214;-$877)				
**Longer duration of RHD (fewer YLLs)**	-$2305	100%	100%	100%	100%
	(-$4520;-$1205)				
**Ten-fold higher program cost**	-$732	94.2%	96.2%	100%	100%
	(-$1875; $192)				
**Worst case (all the above changes)**	-$722	84.9%	88.2%	100%	100%
	(-$2305; $725)				

Acceptability thresholds: “best buy” is < $100/DALY; “very cost-effective” is < $5702/DALY; “cost-effective” is < $17,106/DALY. All costs in 2010 US dollars. See main text for details of each scenario. ICER = incremental cost-effectiveness ratio.

In terms of acceptability thresholds, we used the average health outcomes and savings in direct medical costs to calculate the acceptable program cost under the base and worst case scenarios. In the base case, any program costing less than US$ 8.6 million over 10 years would be a “best buy,” and any program costing less than US$ 36.9 million and US$ 94.5 million would be “very cost effective” and “cost effective,” respectively. The analogous program cost thresholds in the worst case scenario were US$ 341,500, US$ 19.5 million, and US$ 58.4 million over 10 years, respectively.

## Discussion

We report the first economic evaluation of a comprehensive program to prevent and control ARF/RHD in a developing country setting. We found that the program dominated a “do-nothing” approach and was cost saving in the base case scenario. The program also remained cost-effective in several scenario analyses, including a 20-fold increase in program costs. There are three key findings of our analysis that are highly relevant beyond the Cuban population.

Firstly, our analysis suggests that previous cost-effectiveness studies of ARF/RHD control [5, 9, 10] might have missed a crucial element: synergy between primary and secondary prevention. In the Pinar del Rio program, the absolute risk reduction in new diagnoses of ARF (i.e., effect of primary prevention) was observed to be 83%, and the reduction in recurrent attacks of ARF (i.e., effect of secondary prevention) 94%. The combination of interventions likely led to a larger and more rapid reduction in the total burden of ARF and RHD in the population (and expenditures on these conditions) than would be observed with selective implementation of either primary or secondary prevention in isolation. This combined approach makes sense from an infectious disease dynamics standpoint, yet it has not been represented in studies that have only considered the choice between primary and secondary prevention (and in some cases, cardiac surgery).

Secondly, we demonstrated that the program’s cost (US$ 202,890) was modest in comparison to acceptable levels of expenditure using standard cost-effectiveness thresholds. Large savings in direct medical expenditures on advanced medical and surgical care were the drivers of our high acceptability thresholds. Thus, while RHD is an uncommon disease (even in endemic countries), it carries a high health and economic burden that is almost completely preventable with existing technologies.

These program costs provide an important starting point for planning ARF/RHD control programs in other settings. The cost of the intervention in Pinar del Rio was about US$ 0.07 per at-risk child per year, which is comparable to the cost of recommended interventions for other neglected tropical diseases, e.g., mass drug administration for lymphatic filariasis [26]. Again, like mass drug administration for parasitic diseases, and in contrast to pharmacologic treatment of many other chronic non-communicable diseases, this cost represents an up-front investment that could lead to better control of the condition in the long run [12].

Thirdly, the findings of this study have immediate implications for policymaking in endemic regions such as sub-Saharan Africa, South Asia, and the Asia Pacific region. In these settings, scale-up of RHD programs is often hindered by lack of recognition of RHD as an important public health problem, political barriers, and budgetary constraints. The results of our analysis suggest that, even in low-resource settings, inaction on RHD is much more costly than action. Furthermore, it is possible that investments in ARF/RHD control could have synergies with other global and local initiatives to strengthen the delivery of primary care, particularly to children [27].

The Cuban experience demonstrated that ARF/RHD control could be feasibly integrated into existing public health services. In other settings, investments in ARF/RHD control, such as (1) improvements in supply chains of penicillin, (2) health care provider training on management of a common outpatient condition (pharyngitis), and (3) development of disease registers, could have a spillover effect and contribute more broadly to health system strengthening in low-resource settings [28]. In this sense, low cost, highly effective ARF/RHD initiatives represent a “best buy” in global health and should be added to WHO’s list of highest-priority interventions for non-communicable diseases [23]. One example of such an initiative is the Stop Rheumatic Heart Disease A.S.A.P. Programme that has been proposed by the Pan-African Society of Cardiology and supported by the World Heart Federation [27, 29].

Our study has some important limitations. First, we did not have access to individual-level data over the study period and thus had to estimate transition probabilities from aggregate data. Second, we did not have a true counterfactual comparator, but rather global and regional trends. Third, the fact that the program occurred over 20 years ago in a centrally planned economy makes an accurate cost assessment challenging. Most relevant to developing countries in the present day, the labor costs in Cuba were inexpensive during the study period (about $1 per hour on average), and thus the costs might not be generalizable to settings with higher wages. On the other hand, conversion of Cuban Pesos to US dollars and use of medical costs charged to foreigners may over-estimate the cost denominator. We did, attempt to address all these limitations in the scenario analyses, and our findings were substantively similar across a wide range of assumptions.

The results of this study should be interpreted with some caution. In addition to the caveats above, we should note that there was full access to cardiac surgery in Cuba during the Pinar del Rio program; thus it is possible that the case for prevention in Cuba (especially as a cost-saving measure) could be much stronger than, e.g., in a very resource-limited area without surgical access. On the other hand, we were unable to capture the broader health system effects of the Cuban program in our model. As discussed above, it is possible that such effects could make a strong case for prevention, even without the additional savings in surgical expenditures.

Despite these limitations and caveats, our analysis suggests three important directions for future research. Firstly, the results of this program, as well as similar programs in the Caribbean [13, 30], have not been replicated since the late 1990s. Assessments of the effectiveness of ARF/RHD programs currently underway [27] will provide more relevant inputs and assumptions for future economic evaluations. Secondly, future cost-effectiveness studies of ARF/RHD prevention should include scenarios where combined primary and secondary prevention are assessed alongside isolated interventions. It is possible that short-term budgetary constraints in very low-resource settings could make large-scale combined preventative or surgical interventions difficult to implement, even if they are highly effective. Finally, it will be necessary to conduct operational/implementation science research on how to optimize delivery of ARF/RHD interventions within the existing healthcare infrastructure. Such research will shed light on the possibilities for health system strengthening through investment in ARF/RHD control.

## Conclusions

We conclude that a 10-year program to control ARF and reduce the burden of RHD in Pinar del Rio, Cuba was cost saving. The results of our analysis were robust to large increases in the program’s cost and more conservative assumptions about the program’s net effects, and in fact, even in the worst case scenario, there is an 88% chance that such a program is a “best buy” in global health. It is likely that the effectiveness of the Cuban program arose from synergistic effects of primary and secondary prevention activities within the comprehensive framework. The findings of this study have important implications for policymaking in other limited resource regions, where similar programs could be undertaken to reduce the health and economic impact of ARF/RHD.

## Supporting Information

S1 File Details of the cost-effectiveness modeling strategy. (DOCX)

S1 Table Full list of transition probabilities used in the Cuba RHD model. (PDF)

S2 Table Program costs (2010 USD) calculated from human resource inputs, typical salaries, and materials. (PDF)

S3 Table Medical costs (2010 USD) calculated from data collection sheets by Lopez (2000) and Nordet et al. (2008). (PDF)
