# Supplementary material for: A Cost-Effectiveness Analysis of a Program to Control Rheumatic Fever and Rheumatic Heart Disease in Pinar del Rio, Cuba
Source: PLoS One. 2015 Mar 13;10(3):e0121363. doi: 10.1371/journal.pone.0121363 (PMC4358951; doi:10.1371/journal.pone.0121363)
Supplement: S1 File — (DOCX) [file pone.0121363.s001.docx]

**S1 File. Details of the cost-effectiveness modeling strategy.**

**Epidemiological and clinical inputs**

The main manuscript describes the key epidemiological inputs (Table 1) used for our decision tree (Fig. 1). In order to estimate long-term outcomes following ARF, we calibrated the decision tree using clinical data from 1986 (“do nothing”) and 1996 (intervention), which allowed us to calculate transition probabilities that captured the program’s effectiveness at reducing recurrent ARF, progression to RHD, and severe/fatal RHD. We also assigned average medical costs to each disease state using local cost data (see below). The final decision tree precisely predicted the observed patient outcomes and predicted medical costs to within 7.1% (intervention scenario) and 6.2% (control scenario) of their published values. S1 Table lists all the probabilities used in our decision tree.

**Program costs**

The main manuscript describes the basic components of the program: administration, education, surveillance, and external evaluation. The administrative staff included a part-time program manager, 14 part-time local medical representatives who were involved in the educational activities, a full-time administrative assistant, and a 16-member ad hoc advisory committee consisting of clinicians and epidemiologists. To account for the operating costs of the program, we used standard hourly wages for Cuban professionals at these levels of training during the 1980s (Ministry of Health – personal communication). We separately accounted for the time spent by local representatives, nurses, and physicians conducting health educational campaigns and individual patient counseling on ARF and RHD.

As described in the main text and by Nordet et al., the program also included community-based prevalence studies before (1985) and after (1996) the educational activities. These studies used auscultation as a screening tool and referred children to a cardiologist for confirmatory echocardiography in the case of a suspicious murmur. We accounted for the cost of screening materials, the survey coordinator’s salary, and the salaries of the physicians conducting the screening or confirmation. WHO’s 1997 salary rates were inflated to 2010 US dollars. The final aggregated program cost estimates are listed in Table 2; individual components are detailed in S2 Table.

**Direct Medical Costs**

The main text describes the process of calculating direct medical costs. We took direct medical cost data from Nordet et al. and Lopez, who produced a series of average costs for patients with ARF and RHD. We used weighted averages in cases where their level of clinical detail was greater than that used in our model. As mentioned above, our decision tree model was very accurate at reproducing the medical costs reported by Nordet et al. in 1986 and 1996. S3 Table lists all the cost estimates used in our model.

**Disability-adjusted life years**

DALYs incorporate both years of life lost (YLL, i.e., premature mortality) and years lived with disability (YLD, i.e., morbidity). For the YLLs, we used life expectancies for Cuba in 1990, and we calculated remaining life expectancies for four 5-year age groups (5-9, 10-14, 15-19, and 20-24 years) using WHO’s life tables. YLDs were calculated as follows: consistent with prior GBD studies, we used the influenza disability weight (0.21) to capture morbidity from ARF (assuming a 14- to 28-day episode). We used disability weights for moderate (0.037) or severe (0.186) heart failure in our chronic heart failure state, and the disability weight for mild heart failure (0.037) in our post-surgical state. All disability weights were taken from the GBD 2010 study.
