# Supplementary material for: A Cost-Effectiveness Analysis of a Program to Control Rheumatic Fever and Rheumatic Heart Disease in Pinar del Rio, Cuba
Source: PLoS One. 2015 Mar 13;10(3):e0121363. doi: 10.1371/journal.pone.0121363 (PMC4358951; doi:10.1371/journal.pone.0121363)
Supplement: S1 Table — (PDF) [file pone.0121363.s002.pdf]

S1 Table. Full list of transition probabilities used in the Cuba RHD model.

State	Do nothing			RHD program		
	base	min	max	base	min	max
ARF 1 (vs. RHD 1)	0.738	0.677	0.799	0.810	0.649	0.970
ARF 1 case-fatality rate	0.014	0.005	0.020	0.014	0.005	0.020
ARF 2 remission rate	0.433	0.365	0.502	0.824	0.668	0.979
ARF 2 (vs. RHD 2)	0.667	0.601	0.732	1.000	0.500	1.000
ARF 2 case-fatality rate	0.029	0.006	0.053	0.000	0.000	0.500
RHD 2 risk of severe disease	0.529	0.460	0.599	0.000	0.000	0.500
RHD 2 case-fatality rate	0.444	0.376	0.513	0.000	0.000	0.500
RHD 2 risk of surgery	0.200	0.145	0.255	0.000	0.000	0.500
RHD 1 risk of severe disease	0.438	0.369	0.506	0.000	0.000	0.500
RHD 1 case-fatality rate	0.357	0.291	0.424	0.000	0.000	0.500
RHD 1 risk of surgery	0.111	0.068	0.155	0.000	0.000	0.500
ARF 3 remission rate	0.278	0.216	0.340	0.750	0.573	0.927
ARF 3 (vs. RHD 3)	0.000	0.000	0.058	0.000	0.000	0.500
ARF 3 case-fatality rate	0.000	0.000	0.058	0.000	0.000	0.500
RHD 3 risk of severe disease	0.929	0.893	0.964	0.000	0.000	0.500
RHD 3 case-fatality rate	0.385	0.317	0.452	0.000	0.000	0.500
RHD 3 risk of surgery	0.250	0.190	0.310	0.000	0.000	0.500

Numbers refer to the position of each disease state within the decision tree (see Fig. 1, main text). Generally, ARF 1 represents primary presentation with ARF, and ARF 2 represents recurrence of ARF in a patient with history of ARF. Similarly, RHD 1 represents primary presentation as ARF with RHD, and ARF 3 and RHD 3 represent recurrence of ARF or progression of RHD in a patient with a history of RHD.

The above probabilities reflect the results of calibrating our decision tree (see Fig. 1, main text) to the empirical results reported by Nordet et al. (2008). In that paper, the program was found to be highly effective, leading to a dramatic reduction in the incidence and severity of RHD. Hence, after the intervention, there were essentially no deaths or cases of severe RHD reported, though the sample size was small (n = 6 cases of RHD in 1996). To account for the "measurement uncertainty" associated with so few observed cases of RHD, we calculated confidence intervals for the states where the transition was reported to be 0.00 (or 1.00, in a couple of cases). In these instances, we estimated the standard error as 3/n, to generate a maximum or minimum value. In the probabilistic sensitivity and uncertainty analysis, we randomly drew values from a beta distribution parameterized by these minimum – maximum values, so in most of the draws, the transition probability was non-zero. These uncertainty estimates are presented

as a range of incremental cost-effectiveness ratios in Table 3 of the main text as well as in a scatterplot in Fig. 2 of the main text.
