# Supplementary material for: A Cost-Effectiveness Analysis of a Program to Control Rheumatic Fever and Rheumatic Heart Disease in Pinar del Rio, Cuba
Source: PLoS One. 2015 Mar 13;10(3):e0121363. doi: 10.1371/journal.pone.0121363 (PMC4358951; doi:10.1371/journal.pone.0121363)
Supplement: S2 Table — (PDF) [file pone.0121363.s003.pdf]

S2 Table. Program costs (2010 USD) calculated from human resource inputs, typical salaries, and materials.

ADMINISTRATIVE COSTS

see cost sheet part 1

position	number	hours/wk	salary/hr	total cost (10 yr)	inflated cost
program manager	1	8	0.69	2750	5471
advisory committee	16	1	0.69	5500	10943
local medical rep	14	2	0.50	7000	13927
admin assistant	1	40	0.25	5000	9948
logistics per PN					3084
SUBTOTAL					**43372**

EDUCATIONAL COSTS - HEALTH WORKERS

see cost sheet part 5

per PN: 2 lectures/year x 14 municipalities = 28 lectures/yr	cost/yr	total cost (10 yr)	inflated cost
thus, cost of each lecture + logistics = US$ 200 -->	5600	56000	111415
educational materials	2000	20000	39791
SUBTOTAL			**151206**

EDUCATIONAL COSTS - COMMUNITY

see cost sheet part 6

	hr spent/pt	# pts	pt-hrs	salary/hr	total cost (10 yr)	inflated cost
RN education 2ppx	1	193	193	0.31	603	1200

MD education 2ppx	0.083	193	16.08	0.50	80	160
SUBTOTAL						**1360**

(education assumes 5 minutes spent w/ patient and family each visit; RN visits once monthly, MD visits once yearly)
(radio/TV spots: no opportunity cost of broadcasting since no competing ads in 1990s… prep time cost is included in admin costs above)
(posters in health centres and schools: time included in admin costs, materials included in education/HW costs)

SURVEILLANCE COSTS
see cost sheet part 7

1986 survey	screened	number/hr	pt-hrs	MD sal/hr	RN sal/hr	AA sal/hr	total cost	inflated cost
pop screen	5708	8	713.5	0.38	0.31	0.25	669	1331
doubt	1222	2.5	488.8	0.38	xxx	xxx	183	365
probable (cardiol)	122	2	61	0.50	xxx	xxx	31	61
prob coordination	xxx	xxx	80	xxx	xxx	0.25	20	40
SUBTOTAL 1								**1796**

(for "doubt," 2 MDs screened, thus while real #/hr is 5, I divided by 2 as though it took 2x time to screen)

1996 survey	screened	hr spent/pt	pt-hrs	MD sal/hr	RN sal/hr	AA sal/hr	total cost	inflated cost
pop screen	25519	0.083	2127	0.50	xxx	xxx	1063	2115
doubt	8268	0.167	1378	0.50	xxx	xxx	689	1371
prob (cardiol) - R1	1582	0.167	264	0.50	xxx	xxx	132	262
prob (cardiol) - R2	298	0.5	149	0.50	xxx	xxx	75	148
prob coordination	xxx	xxx	200	xxx	0.31	xxx	63	124
SUBTOTAL 2								**4021**

(for "doubt," 2 PCPs screened, thus while real #/hr is 5, I divided by 2 as though it took 2x time to screen)
(for "probable" some pts required full examination thus R2 = round 2)

EXTERNAL EVALUATION COSTS -- ONCE-OFF
see cost sheet part 8

position	number	hours	salary/hr	total cost (10 yr)	inflated cost
national cardiologist	1	16	0.69	11	22
logistics per PN					4
WHO staff	1	24	34.0	816	1108
SUBTOTAL					**1134**

GRAND TOTAL
202890
