# Supplementary material for: A Cost-Effectiveness Analysis of a Program to Control Rheumatic Fever and Rheumatic Heart Disease in Pinar del Rio, Cuba
Source: PLoS One. 2015 Mar 13;10(3):e0121363. doi: 10.1371/journal.pone.0121363 (PMC4358951; doi:10.1371/journal.pone.0121363)
Supplement: S3 Table — (PDF) [file pone.0121363.s004.pdf]

S3 Table. Medical costs (2010 USD) calculated from data collection sheets by Lopez (2000) and Nordet et al. (2008).

PRIMARY PREVENTION COSTS

see cost sheet part 2

	1986	1996	
strep cases	17940	15945	
PHC cost, USD 2000	20	20	
PCN cost, USD 2000	3.82	3.82	
yearly cost	17964	15969	
10-yr cost	179638	159691	
inflated cost	227488	202228	<-- these costs should be added to the fixed program costs

SECONDARY PREVENTION COSTS <-- includes: PHC and cardiology visits, treatments (abx etc), echo

see cost sheet part 4

	listed cost	inflated cost	yearly	compliance weights		1986	1996
years 0-1 (2)	1379.5	1747	873.48				
years 2-7 (6)	2301.96	2915	485.86	regular	1	0.5	0.937823834
years 8-19 (12)	2463.48			irregular	0.5	0.365384615	0.062176166
8-19 yearly	205.29		259.98	none	0	0.134615385	0
thus, years 8-9 (2)	410.58	520					
SUBTOTAL 10-yr		5182		weighted prop tot cost		0.682692308	0.968911917

1986 avg pt cost	3538
1996 avg pt cost	5021

ACUTE TREATMENT COSTS

see cost sheet part 3

	years 0-1	years 2-7	years 8-19	2-yr est	10-yr total	inflated cost	yr 1 hosp	inflated cost
pure ARF	1703.28	1005.96	xxx	335.32	3044.56	3855.54	1023.78	1296.48
ARF/carditis, no seq	2009.2	1005.96		335.32	3350.48	4242.95	1329.70	1683.89
light RHD	2809.2	2301.96	2463.48	410.58	5521.74	6992.57	1429.70	1810.53
mod-severe RHD	3327.6	2301.96	2463.48	410.58	6040.14	7649.05	1948.10	2467.02
mod-sev w/ HF	6802	3445.38	5422.32	903.72	11151.1	14121.42	5009.18	6343.48
surgical cases no HF	18362.88	2581.56	3946.68	657.78	21602.22		16694.18	21141.01
surgical cases w/ HF	18984.92	12126.6			24374.52	29111.78	16694.18	21141.01

(for surgery, weighted costs w/ and w/o HF 50-50)

TOTAL COSTS (weighted 50-50)		w/ 2ppx 86	w/ 2ppx 96	recur hosp
1 - ARF	4049			1490
2 - RHD mild/mod	7321	10859	12342	2139
3 - RHD severe	14121	17659	19142	6343
4 - RHD surgery	29112			
